# The incomparable fascination of comparative physiology: 40 years with animals in the field and laboratory

**DOI:** 10.1007/s00359-023-01681-3

**Published:** 2023-11-21

**Authors:** Horst Bleckmann

**Affiliations:** Institute of Zoology, University of Bonn, Poppelsdorfer Schloss, Bonn, Germany

**Keywords:** Comparative physiology, Lateral line, Hydrodynamic reception, Electroreception, Infrared reception, Peregrine falcons, Biomimetic

## Abstract

This paper is not meant to be a review article. Instead, it gives an overview of the major research projects that the author, together with his students, colleagues and collaborators, has worked on. Although the main focus of the author’s work has always been the fish lateral line, this paper is mainly about all the other research projects he did or that were done in his laboratory. These include studies on fishing spiders, weakly electric fish, seals, water rats, bottom dwelling sharks, freshwater rays, venomous snakes, birds of prey, fire loving beetles and backswimmers. The reasons for this diversity of research projects? Simple. The authors’s lifelong enthusiasm for animals, and nature's ingenuity in inventing new biological solutions. Indeed, this most certainly was a principal reason why Karl von Frisch and Alfred Kühn founded the Zeitschrift für vergleichende Physiologie (now Journal of Comparative Physiology A) 100 years ago.

## Introduction

This special issue is published on the occasion of the 100th anniversary of the Zeitschrift für vergleichende Physiologie (now “Journal of Comparative Physiology A”). It is also a clear statement of the importance of comparative research. I already had my first encounter with comparative biology as a student. In the animal physiology course, we had to prepare for each experiment. One of the textbooks that I used for my preparation was “Animal Physiology” from Heinz Penzlin. This textbook immediately captivated me because it was truly comparative. From Penzlin’s textbook I learned, for example, that the morphology and physiology of lungs, kidneys, brains and sensory organs can be very different in different animal species because different species live in different environments and thus are exposed to different selection pressures. My early enthusiasm for comparative biology finally led me to revise, together with Jan Hildebrand and Uwe Homberg, the “Penzlin” more than four decades later (Hildebrandt et al. 2020). As a student of biology, I also learned from the “Penzlin”—among others—that one can fully understand the morphology and physiology of an animal only if one considers its evolution and the natural environment in which this particular animal must function, i.e., if one does comparative research. No wonder that I submitted my first scientific manuscript to the “Zeitschrift für vergleichende Physiologie”. This journal, founded by Karl von Frisch and Alfred Kühn in 1924, was one of the few journals that at that time already emphasized the importance of the comparative aspects of physiological research. To my relief, my first manuscript was—after taking the suggestions of two reviewers into account—accepted for publication (Bleckmann 1980). This paper would be the first of 39 papers which I submitted and published in the Journal of Comparative Physiology A.

This article is not meant to be a review article of all my past work. Instead, I will summarize some of the various research projects we did in my laboratory but also how these projects came about. Since I recently have published an article about my lateral line research (Bleckmann 2023), this paper especially gives information about all the other research projects that I, together with my students, colleagues and collaborators have worked on. As the reader will learn, it was typically a combination of chance, curiosity and grant money that determined who I cooperated with and which animals’, respectively, scientific questions I investigated.

I wrote my diploma and doctoral thesis in the laboratory of Erich Schwartz from the University of Giessen. He and his students investigated the sensory basis of lateral line-mediated prey capture behavior in surface feeding fish (Schwartz 1965). With their cephalic lateral line, these fish detect and localize terrestrial insects trapped at the water surface. In the laboratory of Erich Schwartz, I already did my first comparative study. Among others, I found that both, the topminnow *Aplocheilus lineatus* and the butterflyfish *Pantodon buchholzi*, determine the distance to the center of a concentric surface wave by analyzing the frequency content, frequency modulation and curvature of the wave train. My studies were the first to show that two distantly related fish, although equipped with completely different cephalic lateral lines, do the same stimulus analysis for prey detection and prey localization. Furthermore, I uncovered that both species use all the hydrodynamic (physical) information theoretically available to determine the distance a surface wave stimulus has traveled (Bleckmann and Schwartz 1982; Hoin-Radkovski et al. 1984; Bleckmann 1988a).

### From fish to fishing spiders

In spring 1981, 1 year after my doctorate, Friedrich Barth from the University of Frankfurt offered me a postdoctoral position. The reason probably was that he was looking for someone who was familiar with the physics of water surface waves and had the motivation to study fishing spiders. Barth, who—for many years—served as the Editor in Chief for the Journal of Comparative Physiology A, already was an internationally recognized scientist who studied mechanosensation in spiders and spider behavior. He suggested that I should investigate the sensory basis of prey capture behavior in the fishing spider *Dolomedes triton*. Even though I would have to move from animals with backbones to invertebrates, I would stay with mechanoreception and the aquatic environment. This made my move from Gießen to Frankfurt easy.

**Fig. 1:**
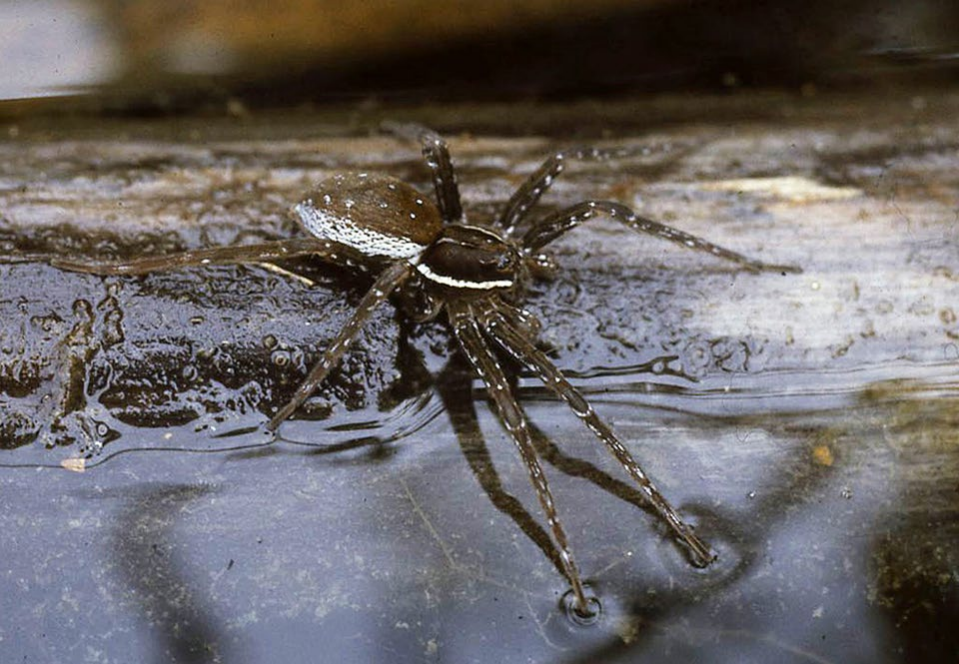
The fishing spider *Dolomedes trition* in lurking position at the edge of a pond

Nearly all over the world, fishing spiders inhabit small ponds and the shoreline of lakes (Bleckmann and Rovner 1984). Like surface feeding fish, these spiders respond to capillary surface waves with a directed turn and a subsequent forward motion toward the wave source. One of the first things which struck me was the behavioral difference between surface feeding fish and fishing spiders, although both occupy the same ecological niche. Surface feeding fish tend to move around and show “emotions” (e.g., by rapidly oscillating their breast fins if frightened). In addition, these fish not only can learn, but also remember what they have learned over many weeks. Fishing spiders were different: for many hours, *Dolomedes* sat motionless at the edge of the experimental tank. While in ambush, up to four legs touched the water surface (Fig. 1). Since spiders cannot be conditioned, I relied on their innate behavior. I was somewhat frustrated that *D. triton* rarely responded to the wave stimuli I presented, but when it did so the response was usually very fast.

My research project in Frankfurt was part of a comparative study in which the sensory basis of prey detection in fishing spiders, web spiders and in spiders hunting on solid substrates was investigated by various laboratory members. I found that, unlike surface feeding fish, *D. triton* uses only the curvature of the wave front for distance determination (Bleckmann and Barth 1984). The large leg span of fishing spiders (about 4 cm) no doubt makes it easier for them to determine the curvature of a concentric wave stimulus. Many years later, my student Martin Borchard showed that the same holds true for the fishing spider *D. okefinokensis* (Bleckmann et al. 1994). I also found out how *Dolomedes* discriminates prey waves from non-prey waves, i.e., waves caused by wind, water drops or falling leaves. For this task, *Dolomedes* analyzes the frequency content, time structure and duration of a surface wave stimulus (Bleckmann 1985). Fishing spiders even weigh the amplitude–frequency content of a stimulus with respect to the distance the stimulus has traveled, taking the low pass filter properties of the water surface into account (Bleckmann 1988b). This shows that their stimulus-analyzing capabilities are as sophisticated as those of surface feeding fish. Thus, during my time in Frankfurt I learned for the first time that the small brains of invertebrates can perform as well as the much larger brains of vertebrates. In Barth’s laboratory, I was also involved in a study of the terrestrial wandering spider *Cupiennius salei*, a spider that is native to Central America and lives on banana plants and bromeliads. In a field study in Mexico, we measured the substrate-borne vibrations the spiders are typically exposed to when sitting on their dwelling plants. As *Dolomedes*, *Cupiennius* uses the frequency content, time structure and duration of a stimulus to discriminate prey from non-prey substrate vibrations (Barth et al. 1988a, 1988b). In retrospect, considering how orb-weaving spiders are adapted to read the vibrations of an insect trapped in a web (Klärner and Barth 1982), it is not surprising, given the diversity of spiders, that they would exploit this vibrotactile ability for any number of other prey capture scenarios.

Within the first 2 years after my PhD, Jochen Autrum, then Editor in Chief of the Journal of Comparative Physiology A, asked me to review a manuscript. I felt honored and consequently put a lot of effort into my review. Of course, the result was that I immediately received more manuscripts to review. Autrum did not mince words and very directly chastised me when the first manuscript I submitted to his journal was missing statistics. As a result, I got into the habit of always backing up my data with thorough statistics. One day, Autrum sent me the reports of the two referees on another manuscript I had submitted. In his accompanying letter, he wrote “There are two cases of stupidity. In the first case authors do not do the necessary statistics and in the second case they statistically back up data that are so clear that no statistic is needed.” I had to admit that Autrum was right.

In spring 1985, I moved to the Scripps Institution of Oceanography in San Diego (La Jolla, CA), to work in the laboratory of Theodor Holmes (Ted) Bullock, one of the most prominent comparative neurobiologists at the time. Although my spider studies had been very interesting I decided to return to the fish lateral line. One of my goals was to learn how to record from central lateral line units. Instead of working with teleost fish, we decided to work with cartilaginous fish. The reason was simple: Mechanosense in cartilaginous fishes was very much understudied compared with teleost fishes. At Scripps I recorded from central lateral line units of thornback guitarfish and hornsharks. I found that cartilaginous fishes process lateral line information at all levels of the neuraxis, from medulla to telencephalon. Like other studies (Schweitzer and Lowe 1984; Corwin 1991), my study showed that afferent pathways in the brain of elasmobranchs are much more like those in advanced vertebrates than was formerly believed (Bleckmann et al. 1987, 1989).

Soon after my arrival in La Jolla, I attended the International Conference on “Sensory Biology of Aquatic Animals” at the Mote Marine Laboratory in Sarasota, Florida. At this conference, organized by Art Popper, Dick Fay and others, I listened to a talk given by Uli Budelmann, a zoologist well known for his cephalopod studies. From Uli, I learned that cephalopods have lines of ciliated cells, called epidermal lines, on their head and arms. Without providing physiological evidence, Uli suggested that these lines function as hydrodynamic sensors (Budelmann 1988). Since Uli studied cuttlefish at the University of Texas in Galvaston, I invited him to come to the Scripps Institution of Oceanography to find out whether the epidermal lines of cephalopods are indeed sensitive to water motions. A few month later, when I picked him up from San Diego airport, he had 30 juvenile squids (*Sepia officinalis* and *Lolliguncula brevis*) in his hand baggage. Our initial attempts to get neuronal responses from the epidermal lines failed. But after we had tried out several electrode types, we succeeded in recording microphonic potentials and summed action potentials to single-frequency water motions. Similar to the fish lateral line (Bleckmann and Topp 1981; Bleckmann and Münz 1990; Münz 1989), minimal thresholds to local water displacements were found in the frequency range 75–100 Hz (Budelmann and Bleckmann 1988). The microphonic potentials recorded from the epidermal lines had twice the stimulus frequency, also in agreement with the microphonic potentials recorded from the fish lateral line (Flock 1965). Furthermore, responses were especially prominent at the onset of a stimulus. Our results proved that the epidermal lines of cephalopods are an invertebrate analog to the mechanoreceptive lateral line of fishes. In further experiments we found that the epidermal lines of cephalopods were extremely sensitive to abrupt frequency changes. A change of only 1 Hz, e.g., from 100 to 99 Hz, even at low stimulus amplitudes caused a clear neural response (Bleckmann et al. 1991b).

While in Ted Bullock’s laboratory, Glenn Northcutt was hired by the University of California San Diego. Before I met Glenn in San Diego, I was not especially interested in comparative neuroanatomy, but after several conversations with him, his postdoc Mario Wullimann and his PhD students Georg Striedter, Jaqueline Webb and Jiakun Song, I realized that comparative neuroanatomy is indeed another exciting branch of neurobiology. Now motivated to do some neuroanatomical studies, straight away and together with Eberhard Fiebig, a postdoc from Germany, I traced cell groups afferent to the telencephalon of *Platyrhinoidis triseriata* (Fiebig and Bleckmann 1989). Glenn and I also did a study on the pit organs of the axolotl *Ambystoma mexicanum* (Northcutt and Bleckmann 1993).

**Fig. 2:**
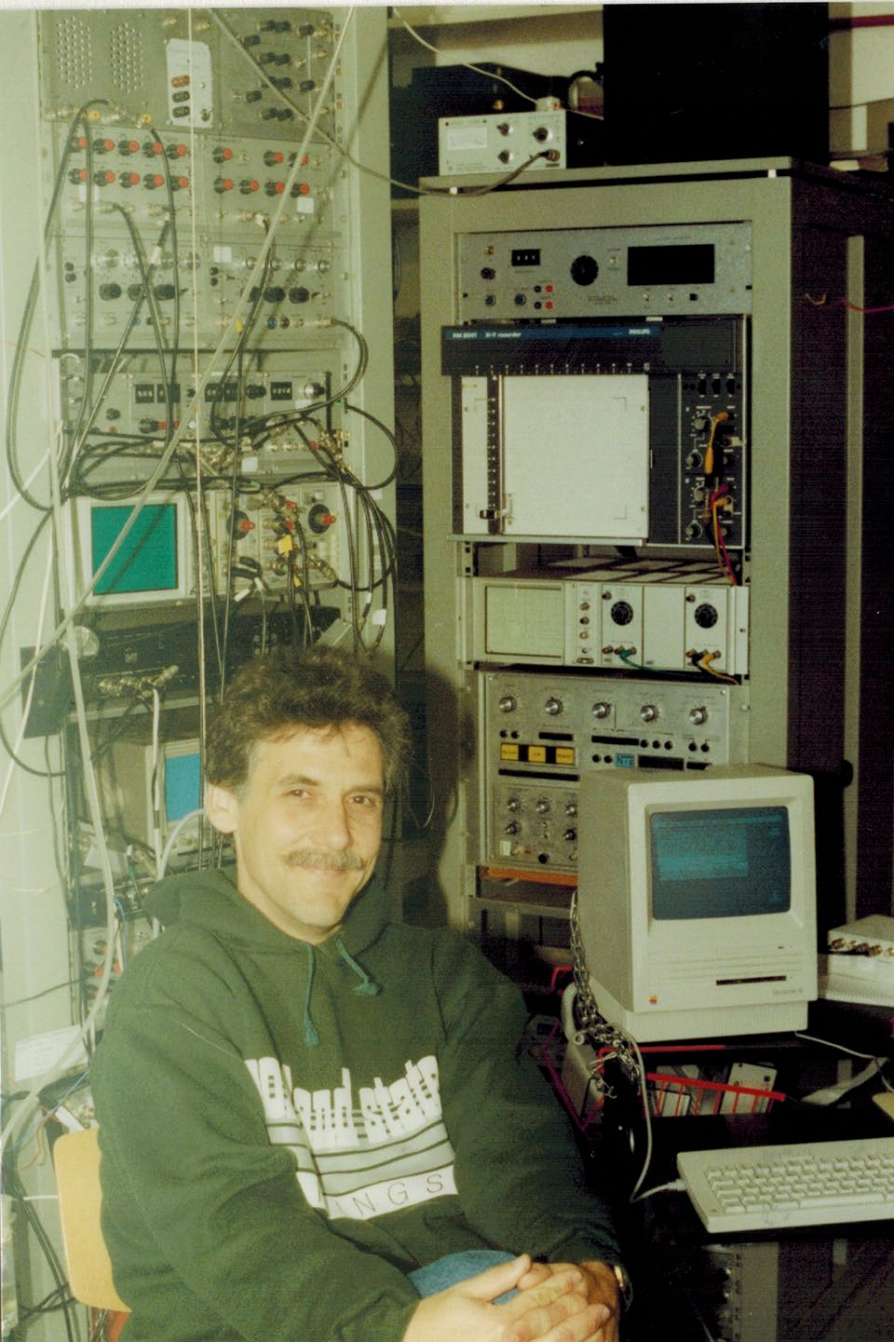
Randy Zelick recording lateral line responses from the weakly electric fish *Eigenmannia* (around 1990). Reprinted with permission of Randy Zelick

While in San Diego I was awarded a Heisenberg fellowship from the German Science Foundation (DFG) which would cover my expenses working in a laboratory of my choice. As it happened, a little later I received an invitation from Peter Görner from the University of Bielefeld (Germany) to work in his laboratory. After my arrival in Germany, I had to decide how to proceed scientifically. Because there were so many open questions still in lateral line research, I continued in this area. Until then, most researchers (including me) had stimulated the lateral line with mono-frequency water motions generated by a sphere that oscillated around a fixed position. That was a clean, but unfortunately very unnatural stimulus. Therefore I, Randy Zelick (Fig. 2) from Portland State University, and my PhD student Horst Müller, decided to stimulate the lateral line with the transient and irregular water motions generated by a small object that passed the fish laterally. Both, peripheral and central lateral line units, responded to such a stimulus. One exciting result was that some midbrain lateral line units were highly sensitive to the direction of object motion (Müller et al. 1996; Bleckmann and Zelick 1993). Since not much was known about the amplitude and frequency content of natural lateral line stimuli I measured, together with Thomas Breithaupt and Reinhard Blickhan, the water motions caused by rapid body or fin movements of fish, frogs and crustaceans. Furthermore, we characterized the water motions in the wake of a trout holding station in a flow tank. Our measurements revealed, that the recorded hydrodynamic events were mostly low frequency, but frequencies up to 100 Hz also occurred (Bleckmann et al. 1991a; Blickhan et al. 1992). Although low-frequency hydrodynamic events dominate in aquatic habitats (Kalmijn 1988), our experiments revealed that high-frequency water motions also occur under natural conditions. This made it clear why the fish lateral line not only responds to low-frequency, but also to high-frequency water motions.

**Fig. 3:**
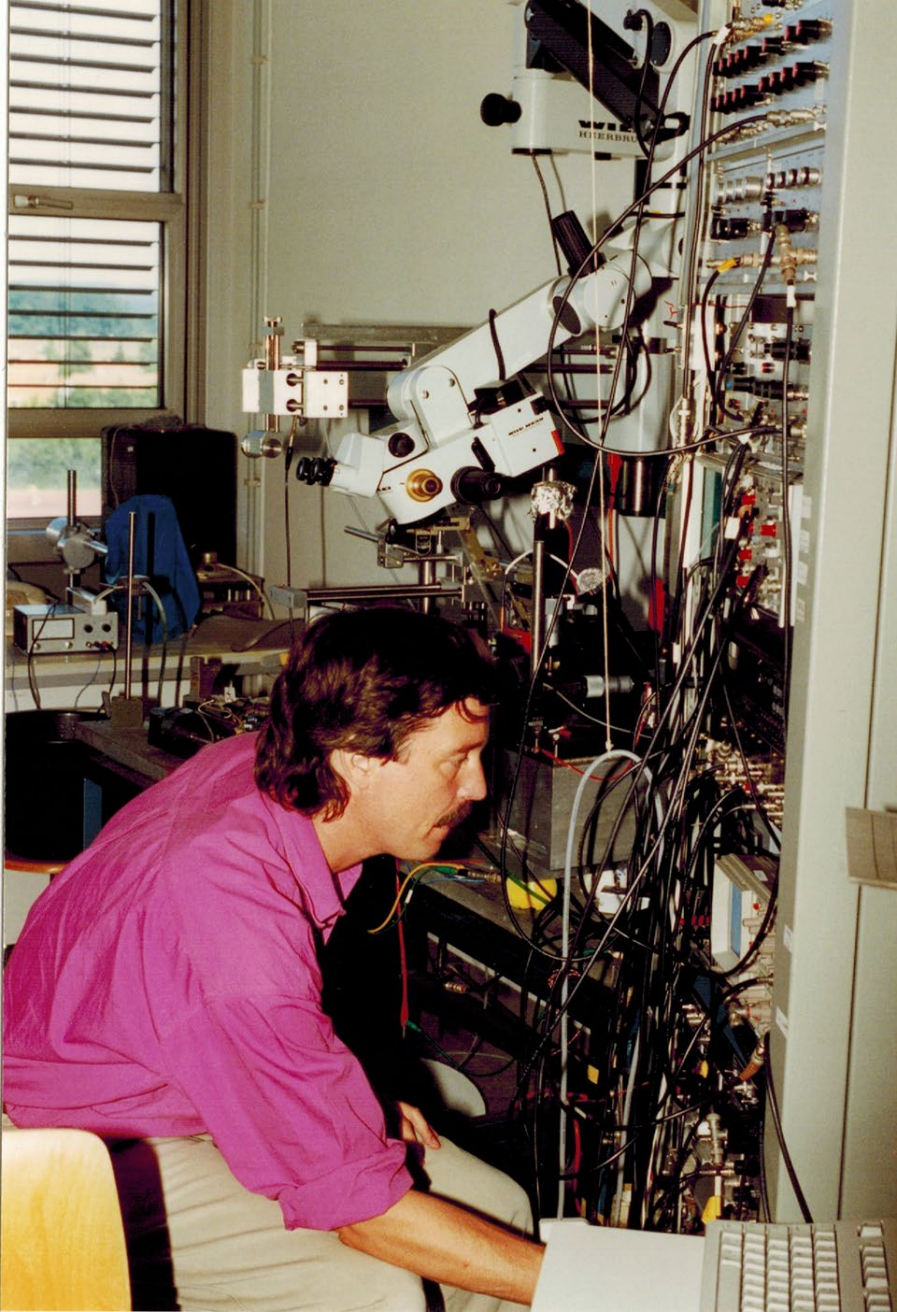
Gerhard von der Emde during our physiological experiments on the phase sensitivity of *Gnathonemus* in 1994. Reprinted with permission of Gerhard von der Emde

Soon after my arrival in Bielefeld, I attended the Göttingen Neuroscience Meeting, organized by Norbert Elsner, his postdocs and students. While in Ted Bullock’s laboratory, I often had met Walter Heiligenberg (who unfortunately died in a passenger plane crash in1994). Walter and his students investigated the neural basis of the jamming avoidance response of weakly electric fish at the level of single cells. Since my encounter with Walter, I also had developed an interest in the electro sense. This was somewhat a natural extension of my research because, from an evolutionary perspective, the electrosensitive lateral line is a derivative of the mechanosensory lateral line. Strolling through the foyer of the conference building I met Gerhard von der Emde. Gerhard (who later became professor at the University of Bonn) presented a poster on the electric fish *Gnathonemus petersii*, and the work he was doing seemed to be very exciting. Like many weakly electric fish, *G. petersii* emits short electric pulses (electric organ discharges or EODs) day and night. *Gnathonemus* uses its self-emitted EODs for electro-communication, object identification and object localization (Moller and Bauer 1973). Depending on their electric properties, electrolocation objects alter the amplitude and wave form (phase) of an EOD. *G. petersii* senses its own EODs with specialized mormyromast electroreceptors distributed over most of the head and body surface. Inanimate objects (e.g,. stones) have purely Ohmic properties, whereas animate objects (e.g., other fishes, insect larvae or water plants) have a complex impedance consisting of a resistive (Ohmic) and a capacitive component. From behavioral experiments, Gerhard knew that *G. petersii* can discriminate purely Ohmic from capacitive objects. While Ohmic objects alter only the amplitude and not the wave form (phase) of the EOD, capacitive objects change both components of the locally perceived EOD. Mormyromast electroreceptors have two types of sensory cells, called A and B cells. Gerhard was convinced that either the A or the B cells must be phase sensitive and that *Gnathonemus* uses the response difference between A and B cells to discriminate between purely Ohmic and capacitive objects. Gerhard, who at that time was a postdoc in Bernd Kramer’s laboratory in Regensburg, had no access to an electrophysiological setup. Being interested in expending a bit into the domain of electroreception, I invited Gerhard to come work in my laboratory on the project (Fig. 3). A few months after the Göttingen meeting, he arrived in Bielefeld with 20 *Gnathonemus petersii* in his baggage. I was skeptical whether Gerhard’s hypothesis was right, but within 4 days we had evidence that only the B cells, but not A cells, responded to small phase shifts in the local EOD. Although the EODs of *Gnathonemus* have a duration of only 0.3 ms, a phase shift of just 1° was sufficient to alter the neural response (von der Emde and Bleckmann 1992). This was a nice example that showed that you can only ask the right question if you take into account the natural stimuli that a sensory system has to encode. Many researchers (including me) had recorded from primary mechanosensory lateral line afferents, but the responses of these afferents to a vibrating sphere stimulus were boringly similar in all species and fibers so far investigated (Münz 1989). No wonder that the clear response difference between A and B sensory cells was very exciting for me.

Much later, I presented our results to a general audience. After my talk, an elderly man came forward. He identified himself as a retired professor of physics. From him I learned that it is quite difficult for physicists to measure small phase differences. To learn more about the mechanisms used by *Gnathonemus* to detect minute phase shifts, he suggested a series of concrete experiments. We did these experiments (von der Emde and Bleckmann 1997), but unfortunately the experiments did not uncover the cellular mechanism of the extreme phase sensitivity of the B cells of *Gnathonemus*. Today, Gerhard thinks that it may not be the temporal phase shift to which the B cells respond. Instead, it could be the simultaneously triggered opposing amplitude shifts of the positive and negative phase of the local EOD evoked by a capacitive object that may cause the B cells to be more or less depolarized and thus to respond to even the smallest capacitive values (von der Emde et al. 2008; von der Emde and Zeymer 2020). As most biologists probably know, it is not easy to decipher the physical and molecular mechanisms that nature exploits.

In 1993, the University of Bonn offered me a full professorship. This allowed me, together with Joachim Mogdans and my new postdoc Michael Hofmann, to continue and extend my lateral line research. We were asking two main questions: first, what do natural lateral line stimuli look like? Up to now we had only studied the water motions caused by fish at a single point in the water column (Bleckmann et al. 1991a). To get information on the three-dimensional extension of fish wakes, my PhD student Wolf Hanke, in cooperation with Christoph Brücker (an engineer of fluid mechanics from the Technical University of Aachen, now professor at the City University of London) used the method of particle image velocimetry. We found that the wake behind a swimming goldfish shows a clear vortex structure for at least 30 s. Particle velocities significantly higher than background noise could still be detected 3 min after the fish (body length 10 cm) had passed (Hanke et al. 2000). This suggested to us that piscivorous fish (and other piscivorous animals equipped with hydrodynamic sensors) might be able to track fish wakes. We also found that the spatial dimensions of wakes caused by fish that differed in their style of locomotion were not all the same (Hanke et al. 2000; Hanke and Bleckmann 2004). Thus animals equipped with hydrodynamic sensors might not only be able to track fish wakes, but in addition may obtain some information about the originator of these wakes.

Our second question was: how does the peripheral and central lateral line cope with quasi natural lateral line stimuli? Since vortices and vortex streets are an important stimulus in natural waters (Rosen 1959; Blickhan et al. 1992) my PhD students Boris Chagnaud (now professor at the University of Graz) and Jan Winkelnkemper investigated how the peripheral and central lateral line responded to laminar water flow and to flow that contained vortices (Chagnaud et al. 2006, 2007; Winkelnkemper et al. 2018). In other studies, my PhD student Jacob Engelmann (now professor at the University Bielefeld) investigated how the fish lateral line separates signals from noise. Water motions were generated with a vibrating sphere or with an object that passed the fish laterally. Jacob found that the responses of primary afferents that received input from superficial neuromasts were masked in running water. In contrast, the responses of afferents that received input from canal neuromasts were not or only slightly masked (Engelmann et al. 2000, 2002a). This was the first study to show a biologically relevant clear difference in the responses of superficial neuromasts and canal neuromasts. Our study supported the idea that the lateral line of fish that have few superficial neuromasts, but a well-developed canal system is adapted to running water conditions, whereas the lateral line of fish that have only few superficial neuromasts is adapted to stillwater conditions (however, when we compared six stillwater with six running-water European cypriniform fish species, we did not find such a correlation (Beckmann et al. 2010)). In other studies, we investigated how central lateral line units cope with hydrodynamic noise (Engelmann and Bleckmann 2004; Engelmann et al. 2002b, 2003).

Pushing yet further, we investigated how space is represented in the central lateral line system (Voges and Bleckmann 2011; Meyer et al. 2012; Plachta et al. 2003). We not only compared stillwater fish with running-water fish, but also fish which had only few or many superficial neuromasts or which had an anatomical connection between the cephalic lateral line and the inner ear (Bleckmann et al. 1991c). Thus, our lateral line research was truly comparative and therefore entirely in the spirit of the Journal of Comparative Physiology A.

### Infrared reception in insects

Nearly every year, I would attend the meeting of the German Zoological Society. In 1995, this meeting took place in Kaiserslautern. Walking through the poster exhibition hall, a poster that showed a forest fire caught my attention. One of the authors of the poster was Helmut Schmitz from the University of Erlangen. From Helmut, I learned that the pyrophilic beetle *Melanophila acumilata* (Buprestidae) approaches forest fires from large distances and that *Melanophila* most likely can detect the IR radiation emitted by forest fires with a pair of thoracic pit organs. Helmut went on to explain that the bottom of each pit consists of 50–100 sphere-like dome-shaped sensilla, each of which is innervated by the ciliary dendrite of a single nerve cell whose ultrastructure is similar to that of a ciliary insect mechanoreceptor. I knew the literature about infrared (IR) reception in snakes, but I had never heard of any insect capable of sensing IR radiation. According to Helmut, the pit organs of *Melanophila* most likely were sensitive to IR radiation, but physiological evidence was lacking. Since Helmut had no access to an electrophysiological setup, I invited him to come to my laboratory. Within 6 months, we were able to show that the pit organs of *Melanophila* are indeed sensitive to IR radiation, and this was the first physiological evidence of an insect IR receptor. The technique we used was similar to that I had applied to the lateral line neuromasts of the Axolotl *Ambystoma mexicanum* and the epidermal lines of cephalopods (Northcutt and Bleckmann 1993; Budelmann and Bleckmann 1988): the tip of a tungsten electrode was slightly advanced into a single dome-shaped sensillum of *Melanophila*. With this method, we learned that best sensitivity was in the wavelength range 2.8–3.5 μm, i.e., in a range that corresponded to the emission maximum of a forest fire. A stimulus intensity of 14.7 μW cm^−2^ was sufficient to generate a neural response. At higher intensities, a stimulus duration of only 2 ms reliably caused one action potential and in a repetitive stimulus regime receptor potentials showed characteristic fluctuations up to a stimulus frequency of 600 Hz. In crotalid snakes, the IR receptors work like a bolometer, where a thin membrane that is thermally isolated from the surrounding tissue is heated by IR radiation. *Melanophila* turned out to use a different mechanism. The pit organs of this beetle functioned according to a photomechanic principle: IR radiation causes the expansion of the cuticle of the IR-absorbing dome-shaped sensilla, which in turn squeezes the ciliary tip of the mechanosensory cell that innervates the sensilla. Such a photomechanic sensor was not shown before for any biological system (Schmitz and Bleckmann 1997, 1998; Schmitz et al. 1997, 2000a).

**Fig. 4:**
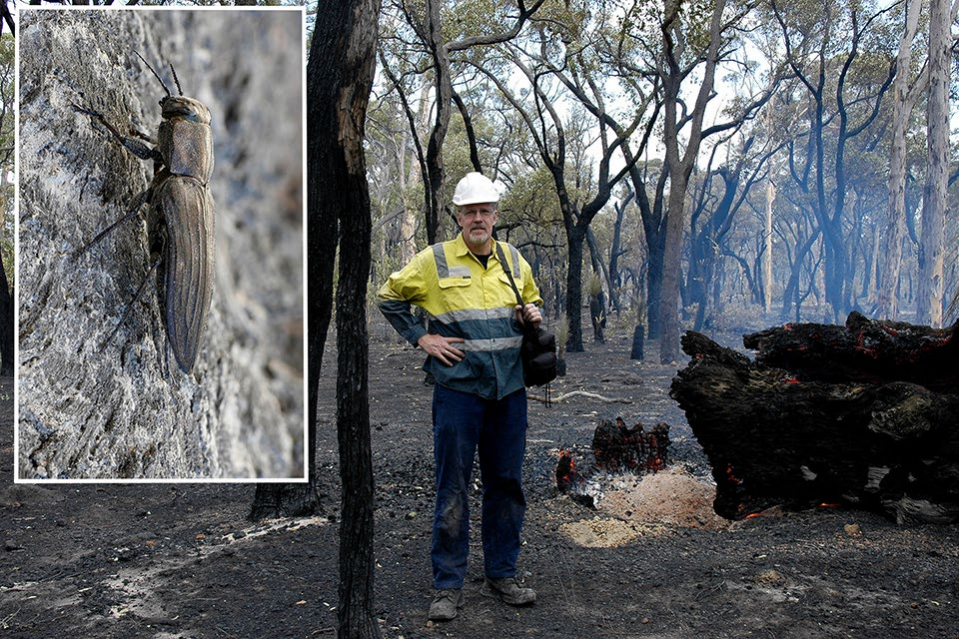
Helmut Schmitz on a burned area in Australia looking for fire loving beetles in 2012. Inset: the pyrophilous beetle *Merimna atrata*. Reprinted with permission of Helmut Schmitz

Helmut and I went on to study other insects capable of forest fire thermoreception. As with *Melanophila*, the Australian beetle *Merimna atrata* (Bupestridae) is attracted by forest fires (Fig. 4). And, again as with *Melanophila*, the larvae of this beetle obligatory depend on the wood of freshly burnt trees. We found (Schmitz et al. 2000b) that *Merimna* has two pairs of IR organs on the ventrolateral sides of its abdomen. However, these organs were fundamentally different from those of *Melanophila*. They consisted of specialized IR-absorbing areas, each of which was innervated by one thermosensitive multipolar neuron. If stimulated with IR radiation, the IR organs of *Melanophila* responded with a short latency in a phasic/tonic fashion. In contrast, the IR organs of *Merimna* compare best with the IR organs of crotalid and boid snakes (Amemiya et al. 1996): IR radiation is absorbed by a special surface and the resulting temperature increase in surface temperature is measured with a thermoreceptor. Most likely, *Merimna* used its IR organs to scan the surface temperatures of burned areas during low-level flight (Schmitz et al. 2000b, 2001). The Australian beetle *Acanthocnemus nigricans* (Acanthocnemidae) is yet another insect attracted by forest fires. Most likely, the larvae of this beetle depend on specialized Ascomycete fungi which become available after a forest fire. *A. nigicans* has a pair of IR organs on the first thoracic segment, each of which consists of a tiny sensory disc which absorbs IR radiation. To reduce heat conduction, each disc is arranged above an air-filled cavity. Inside each disc, about 90 multipolar thermoreceptors are tightly attached to the outside cuticle. Absorption of IR radiation leads to an increase of disc temperature which is sensed by thermoreceptors. The IR receptors of *A. nigicans* can also be best classified as microbolometers with reduced thermal mass (Schmitz et al. 2002). Such receptors resemble the IR receptors of pit vipers (Düring 1974).

Besides fire, warm-blooded animals are a natural source of IR radiation, albeit in a longer wavelength range. The haematophagous bug *Rhodnius prolixus* (Hemiptera) approaches mammals to get their blood (Schmitz et al. 2000c). *Rhodnius* can detect warm air transported by convection and the temperature gradients which occur in the vicinity of a thermal source (Wigglesworth and Gillett 1934). Whether *Rhodnius* can also detect pure IR radiation was discussed by Wigglesworth and Gillett, but experimental proof was lacking. We found that pure IR radiation is sufficient for *R. prolixus* to detect and approach a warm-blooded host (Schmitz et al. 2000c).

### Prey detection in backswimmers

I was involved in the study of two more insect species. Submerged backswimmers (*Notonecta glauca* and *N. maculata*) are covered with a thin air layer (in particular on their hemelytra) that *Notonecta* can hold over a long period of time (Ditsche-Kuru et al. 2011). One day Wilhelm Barthlott, professor emeritus from the University of Bonn, approached me. Preliminary results led him to assume that a thin air layer on the hemelytra was part of a sensory system that *Notonecta* may use for the detection of fish generated water movements. To find out if this is really so, Matthias Mail, Anke Schmitz and Adrian Klein from my laboratory, and others, did an anatomical, histological, behavioral and biomimetic study. We found that the upper side of the hemelytra was hierarchically structured by two types of setae. One type (pin-seta) was tapered and bent, its tip pointed in an anterior-distal direction. The tip of the other type (club-seta) pointed in a posterior direction. With the aid of micro-tomography (μCT) and synchrotron-micro-tomography (SR-μCT), we found that each club and pin of *Notonecta* was in contact with a nerve cell. Thus, as predicted by Barthlott, the clubs and pins most likely were part of a sensory system. Our data suggested that the clubs, which support the air–water interface, are used for hydrodynamic pressure detection. An increase in pressure most likely causes a deflection of the clubs which is detected by the sensory cells that contact each club. The pins protrude from the thin air layer; therefore, the pins most likely are used for the detection of drag caused by water flow. Our data suggest that *Notonecta* can detect hydrodynamic stimuli with their setae by sensing volume changes of the thin air layer and water movements across the hemelytra. Such a sensory principle had never been documented before in any animal. Although we did not obtain physiological evidence, we tested our hypothesis with a homemade biomimetic sensor. With this sensor we were able to record minute pressure changes, for example those caused by human voice (Mail et al. 2018).

### Prey detection in seals

**Fig. 5:**
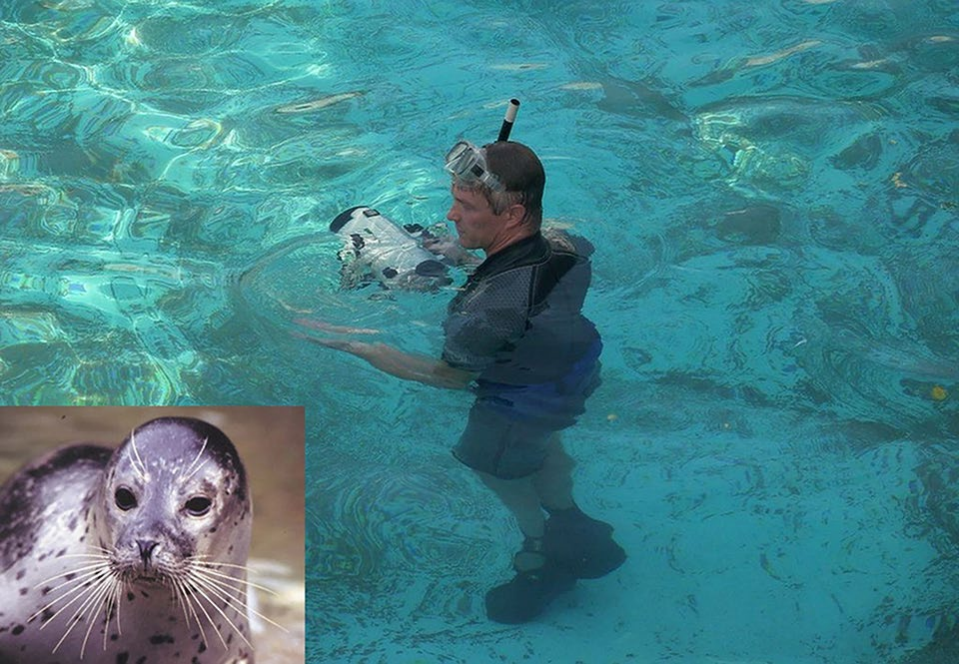
Guido Dehnhard. Inset: harbor seal *Phoca vitulina* (around 1998). Reprinted with permission of Guido Dehnhard

In 1996, Guido Dehnhardt (Fig. 5), at that time already an internationally recognized researcher who studied marine mammals, joined my laboratory. Guido (now Head of the Marine Science Center in Rostock) was already well known for his work on the mechanical sensitivity of the mystacial vibrissae of sea lions (Dehnhardt 1994; Dehnhardt and Drücker 1996), but also for several other studies. Earlier, morphological studies had shown that the vibrissae of seals are strongly innervated (Hyvärinen and Katajisto 1984) and that the vibrissae neuronal information is well represented in the somatosensory cortex (Ladygina et al. 1985). Then, in a set of experiments Guido showed that seals, like rats, use their vibrissae for active touch discrimination and for obstacle avoidance. From Guido, I learned that seals can catch fish even if visibility is reduced, for example at night, in murky waters, or at great depth. Knowing this and given that seals do not echolocate, I had speculated (Bleckmann 1994) that pinnipeds might use their vibrissae for the detection of weak water motions and therefore prey detection. To find out if this is really so, Guido and his PhD student Björn Mauck came to my laboratory where we determined the behavioral threshold of a male harbor seal (*Phoca vitulina*) to the water motions generated with a vibrating sphere. In the test range 10–100 Hz, the seal responded to the water motions with a sensitivity well-tuned to the frequency and amplitude range of fish generated wakes (Dehnhardt et al. 1998).

As already mentioned, we knew that the wake behind a fish of 6 or 10 cm length can last for up to 3 min (Hanke et al. 2000). We also knew that fish wakes show a vortex structure with water velocities well above the threshold of most hydrodynamic receptor systems (Bleckmann 1994; Blickhan et al. 1992). Therefore, piscivorous predators might use fish wakes for long-range prey detection. This suggested to us that seals (and other pinnipeds) may be able to detect and track fish wakes with their facial vibrissae. Together with Björn Mauk, my PhD student Wolf Hanke and Guido Dehnhard, we showed that blindfolded harbor seals—provided their facial vibrissae are free to move—can accurately follow even a curved hydrodynamic trail (Dehnhardt et al. 2001). This finding had solved the mystery how seals find their prey in dark and turbid waters. In a follow-up stud,y we also showed that the Australian water rats, *Hydromys chrysogaster,* can use their facial vibrissae for the detection of water movements (Hanke et al. 2020).

### Spitting cobras, marine snakes and crocodiles

**Fig. 6:**
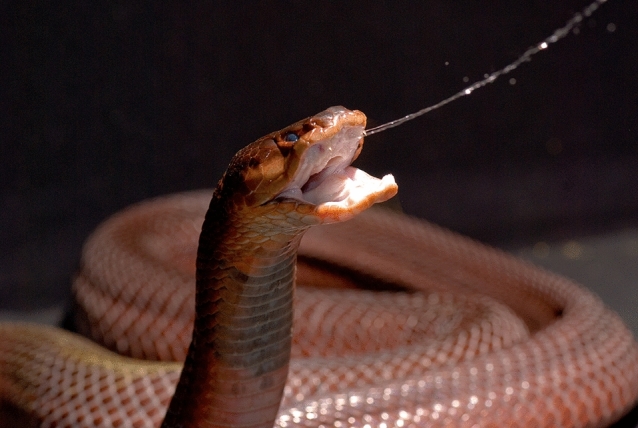
The spitting cobra *Naja pallida* while ejecting its venom. Note that the two venom jets are clearly visible. Photo: Guido Westhoff

In 2000, Guido Westhoff (now Director of the Zoological Garden Hagenbeck in Hamburg), joined my laboratory. Since early childhood, Guido had kept many species of snakes. No wonder that he had an impressive knowledge about snakes and snake behavior. Guido came to my laboratory to study the IR system of crotalid and boid snakes. This resulted in three publications (Ebert et al. 2007; Ebert and Westhoff 2006; Kohl et al. 2012). One day, Guido invited me to his birthday party. In his two-bedroom apartment, he kept many snakes, among others some beautiful spitting cobras. When I rapidly moved my face in front of the terrarium, one of the cobras spat at me. On the glass pane of the terrarium, venom droplets were clearly visible. In popular magazines, I often had read that spitting cobras aim at the eyes of a human harasser. A quick literature search showed that scientific proof for this claim was weak. Therefore, we decided to study the spitting behavior of spitting cobras in more detail. Our student, Katja Tzschätzsch, was eager to do the experiments. We found that spitting cobras (*Naja nigricollis* and *N. pallida*) eject their venom either in distinct jets or in a fine spray if agitated by a human face (in all experiments the face was protected by a transparent visor), the real-size photo of a human face or the life-size face of a doll. During the spitting act (Fig. 6), which lasted for only 70–200 ms, the cobras performed fast undulating head movements that led to a spatial distribution of their venom. To uncover the relevant cues used by cobras for face recognition, my PhD student Ruben Berthe determined how often artificial targets equipped with or without eyes elicited spitting. Oval- and round-shaped targets were most effective, while triangles with the same surface area hardly elicited spitting. The likelihood of spitting depended on neither the presence, the spatial arrangement (horizontal or vertical) nor on the surface texture (shiny or matt) of glass eyes. If a face had two horizontally arranged eyes, the center of the venom droplets was located at eye level between the eyes. Our results indicated that there was no need for the snakes to aim at eyes (Berthe et al. 2013). Provided that the cobra aimed at the middle of the face, the undulating head movements resulted in at least one eye being hit. To ensure that the venom covered the whole face but did not go past the face, the snakes adapted the amplitude of their undulating head movements to face distance, i.e., head oscillation amplitudes decreased if the distance to the human face increased (Westhoff et al. 2005b). Together with Bruce Young from the University of Massachusetts Lowell, we also investigated what part of a human head movement triggered the spitting act. Analyzing over 100 spits, we found that about 200 ms before each spat, Young had suddenly moved his head. To ensure that the moving face was hit, the snakes were accelerating their heads in the same direction as Young’s head was moving. The snakes obviously predicted where the face of Bruce would be at the moment it was hit by the venom (Westhoff et al. 2010).

**Fig. 7:**
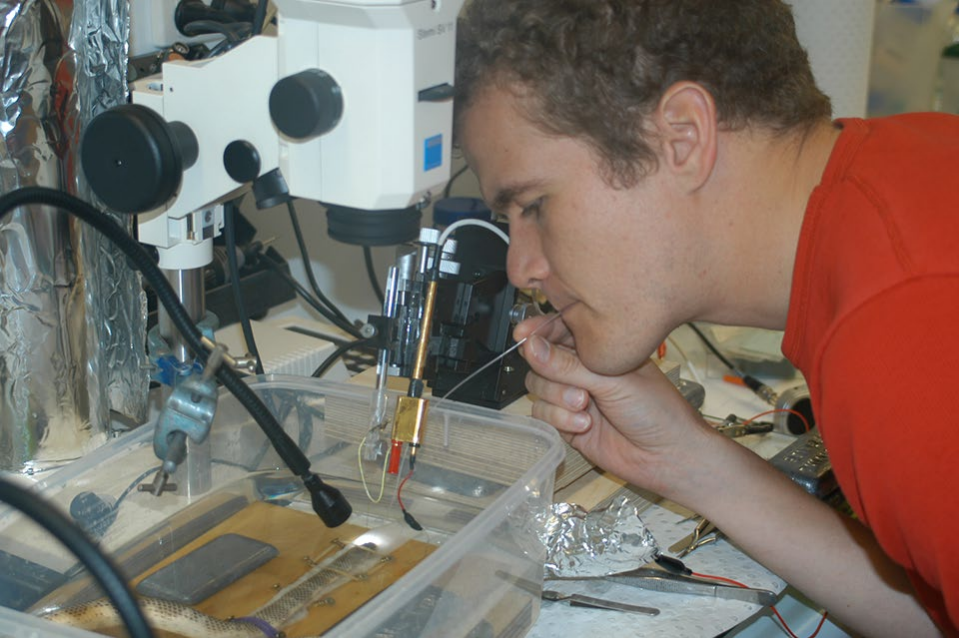
Guido Westhoff giving mouth to mouth ventilation to a sea snake in 2004 in the laboratory of Bryan Frey in Melbourne. Reprinted with permission of Guido Westhoff

Every seventh semester, a German professor gets a sabbatical. Together with Guido Westhoff, I decided to use my sabbatical for the study of sea snakes in Australia. From the literature, we knew that sea snakes (*Lapemis curtus*) and file snakes (Achrochordidae) have scale sensilla which they might use for the detection of mechanical stimuli (Povel and Kooij 1997). This hypothesis was supported by a behavioral study which had found that sea snakes can sense the water motions caused by a swimming fish (Heatwole 1999). Since physiological evidence was lacking, we intended to record nerve impulses from the scale sensilla while stimulating the snakes with mono-frequency water motions. The responses of lateral line afferents can be recorded by slightly piercing the tip of a tungsten microelectrode into the sensory epithelium of a neuromast (Northcutt and Bleckmann 1993). Our plan was to apply the same method to the scale sensilla of sea snakes. In my dream, it would take us 4 weeks to have enough data for a Nature paper, which would leave us 2 weeks for a nice vacation in Australia. We contacted Bryan Fry, a biologist and snake enthusiast from the University of Melbourne (Australia). With Bryan’s help, we caught 30 sea snakes (*Lapemis curtus*) in the Gulf of Carpentaria near Weipa. Catching snakes turned out to be easy because at dusk the snakes came to the warm surface layer of the ocean. Sea snakes were transported by air to the Melbourne Aquarium. During a stopover in Cairns, we were called out over the airport loudspeakers because one of the boxes with the snakes had opened on one side. Since each box carried the label "Caution venomous snakes", the excitement at the airport was great. Fortunately, and in wise foresight, we had placed the snakes inside each box in a second box, so no snake could escape. A few hours later, we landed in Melbourne. Soon after we had figured out how to anesthetize, immobilize and ventilate (Fig. 7) sea snakes the experiments began. Despite our best efforts and the use of many electrode types, we did not get neuronal responses from the scale sensilla. The scale sensilla of sea snakes are innervated by branches of the trigeminal nerve. Thus, the next logical step was to record from the trigeminal nerve fibers that innervated the scale sensilla. When we tried to expose a branch of the trigeminal nerve, we realized that even the slightest cut in the skin caused heavy bleeding that could not be stopped. The reason probably was that sea snakes have skin breathing, i.e., their skin is heavily supplied with blood vessels. From my fish studies, I knew that evoked midbrain potentials can easily be recorded if one stimulates any one of the fish’s sensory systems. Therefore, we tried this method also with sea snakes. On the last 2 days of our stay and after many unsuccessful trials, we finally succeeded in recording evoked potentials from the midbrain of *Lapemis* to water motions generated by a stationary vibrating sphere. In terms of displacement, the lowest thresholds were in the frequency range 100–150 Hz. The sensitivity of sea snakes to water motions was, however, two to three orders of magnitude lower (e.g., 1.8 μm at 100 Hz) than the sensitivity of the fish lateral line (Westhoff et al. 2005a). However, this sensitivity should be sufficient to perceive fish-generated water motions. Unfortunately, our data were not sufficient for a Nature paper. Even worse, since we worked till the very last day no time was left for our planned vacation.

### The hydrodynamic sense of crocodiles

Besides visual, auditory and olfactory cues, crocodiles and caimans use hydrodynamic stimuli for prey detection. Like other surface-dwelling animals, juvenile crocodiles and caimans respond to insect-generated capillary water surface waves which they perceive with integumentary sensory organs, mostly distributed on the cranium (Leitch and Catania 2012). Since threshold values to hydrodynamic stimuli were not known, I suggested to my PhD student Nadja Grap to determine the behavioral sensitivity of juvenile *Crocodylus niloticus* (Crocodilidae) and *Caiman crocodilus* (Alligatoridae) to water surface waves. Nadja found that in the frequency range 40–80 Hz, both species responded to capillary water surface waves, even if water displacement amplitudes were only 1 μm (Grap et al. 2015). In a follow-up study, we showed that juvenile Nile crocodiles and spectacled caimans determine the direction and – to a lesser extent – also the distance a surface wave source. Furthermore, crocodiles and caimans discriminated surface waves that differed only in frequency or frequency modulation (Grap et al. 2020).

### Cognition in sharks, stingrays, cichlids and turtles

**Fig. 8:**
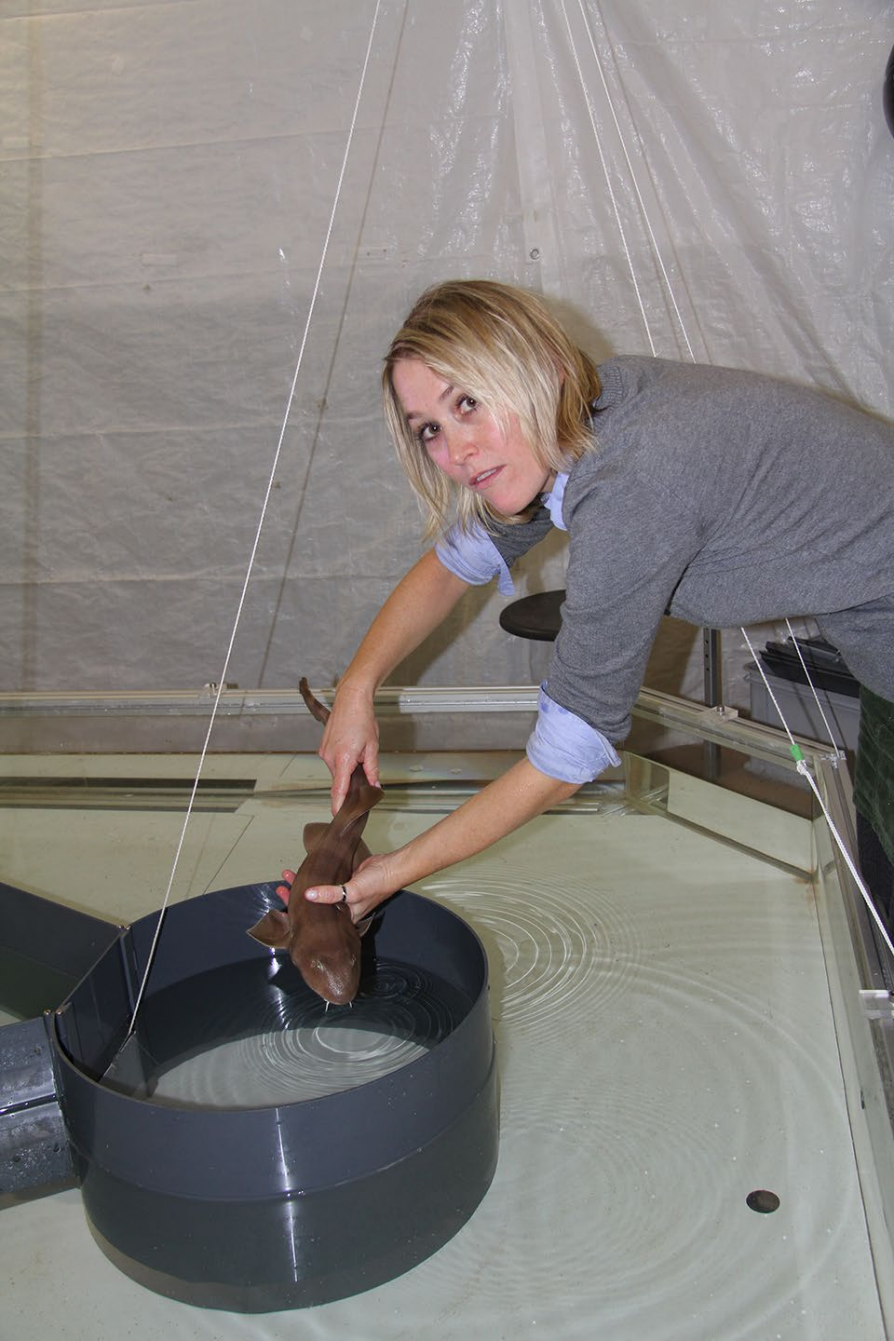
Vera Schluessel while transferring a shark to the experimental tank. Reprinted with permission of Vera Schluessel

As already mentioned, I have been interested in all kinds of animals and animal behavior since my early childhood, so it is not difficult for an unexpected event to pull me into a project with something new. In 2000, Vera Schluessel came to my office. She had obtained her Bachelor of Science at the University of Maryland in the USA and had returned to Germany to continue with her master’s degree. Like me, Vera had always been interested in animals, and in her case especially sharks and shark behavior, which is what she wanted to study for her thesis. Since this was not possible in Bonn at the time, I suggested that she instead studies the orientation strategies of freshwater stingrays. In time-consuming behavioral experiments, Vera found that juvenile freshwater stingrays (*Potamotrygon motoro*) solved spatial tasks by constructing a visual cognitive map of their environment, using allocentric orientation strategies. The rays also learned to use egocentric strategies alone or in combination with allocentric strategies for spatial orientation (Schluessel and Bleckmann 2005). For her doctoral degree, Vera moved to Australia where she studied the life history, population genetics and sensory biology of white spotted eagle rays. After obtaining her PhD, she returned to my laboratory in Bonn. This time she insisted on working on sharks, so we established a seawater aquarium system in our animal holding facility, which allowed us to keep at least small, benthic bamboo sharks. Vera wanted to study the—at this stage almost unknown—cognitive abilities of elasmobranchs in comparison to those of teleost, a much better researched group of fish (Fig. 8). After several saltwater aquaria were finally in place, Vera first continued with her MSc work and investigated spatial strategies and memory retention capabilities in the gray bamboo shark *Chiloscyllium griseum* (Schluessel and Bleckmann 2012). This study was the first to show that sharks, like stingrays and terrestrial vertebrates, can solve spatial tasks and even retain spatial knowledge for an extended period of time. In the many studies that followed, Vera and her students assessed many cognitive skills, ranging from spatial memory and orientation, visual discrimination and categorization of 2D and 3D objects, color vision, serial reversal learning, numerical competence, face recognition, perception of illusory contours, amodal completion and mirror-images, categorization of symmetry, form and size constancy, same–different and matching-to-sample learning, memory retention abilities, avoidance behavior and perception of movement (including biological motion) to acoustic discrimination (Daniel and Schluessel 2020; Fuss et al. 2014a, 2017; Fuss and Schluessel 2015; Gierszewski et al. 2013; Schluessel et al. 2012, 2014, 2015; Schluessel and Düngen 2015). Vera and her students finally repeated these experiments on the turtle *Elseya branderhorsti*, which was found to excel in most of these tasks, often outdoing bamboo sharks, stingrays and cichlids (the data are not yet published). Furthermore, Vera and her PhD student Theodora Fuss showed that in two species of sharks (*Atelomycterus marmoratus* and *Chiloscyllium griseum*), the telencephalon plays a crucial role in place learning, i.e., in allocentric, but not in egocentric orientation (Fuss et al. 2014b, c). Another study identified neural substrates in the telencephalon that are relevant for visual discrimination learning in bamboo sharks (Fuss and Schluessel 2018). In a recent study, Vera and her PhD student Roberta Calvo showed that the inferior lobes of the hypothalamus play an important role in visual learning in cichlids (Calvo et al. 2023).

### From water to air: Peregrine falcons

**Fig. 9:**
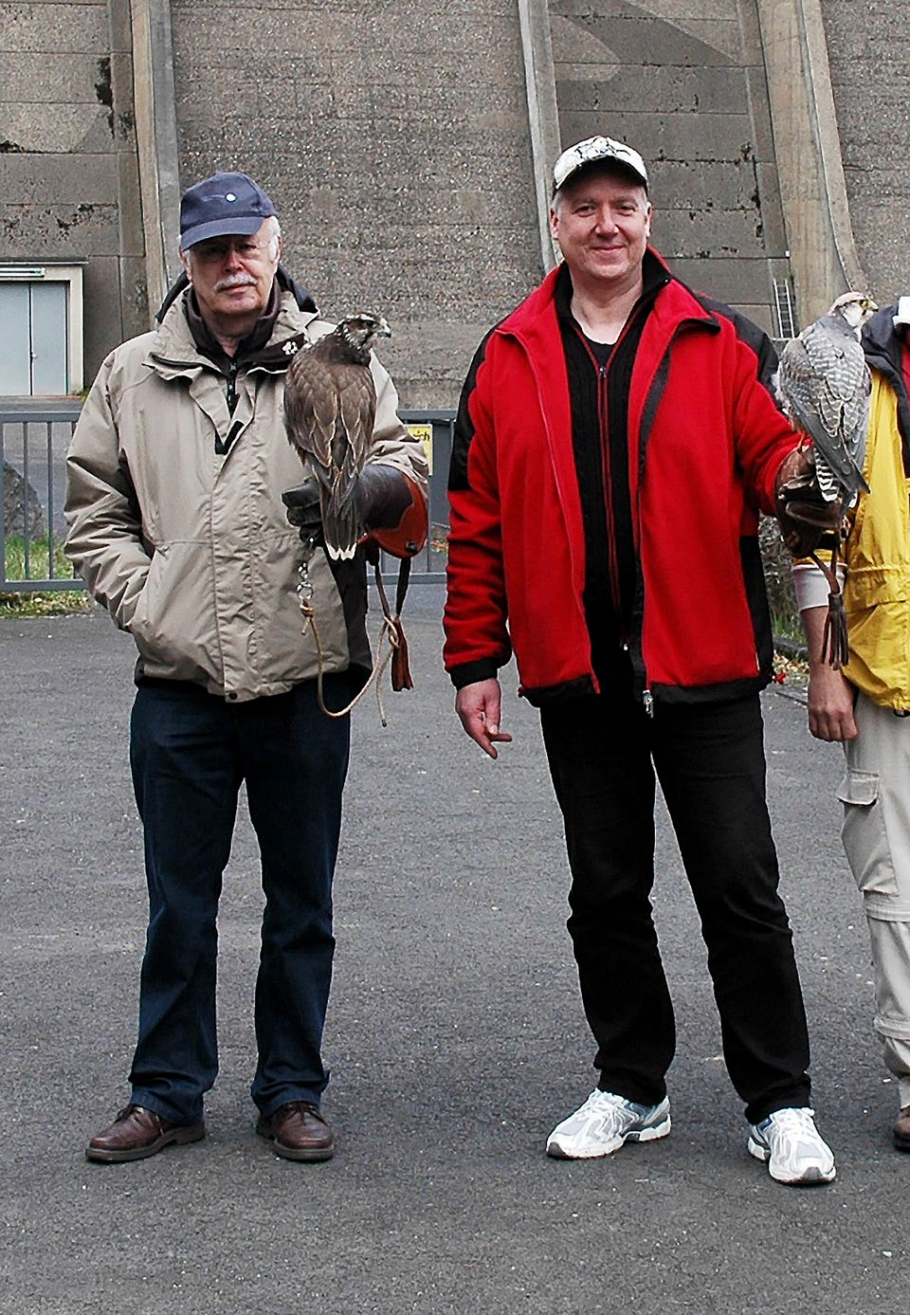
Christoph Brücker (right) and Horst Bleckmann with falcons in front of the dam wall of the Olef Talsperre (Hellenthal, Germany) in September 2012. Reprinted with permission of Christoph Brücker

Why did someone who primarily had studied aquatic organisms work on peregrine falcons? In the 60s of the last century peregrine falcons were threatened worldwide with extinction, their number in Germany was down from about 380 breeding pairs in 1950 to less than 80 breeding pairs in 1965 (Mebs 1969). One of the reasons for this decline was that the fledglings of the few remaining breeding pairs were taken every year from their nesting sites by falconers or pigeon fanciers. To prevent this, volunteers guarded the nesting sites during the whole breeding season around the clock. From 1971 to 1977, I participated in these guards every spring. Peregrines feed on other birds like pigeons and shorebirds that they capture in flight. While attacking a bird in midair, a diving peregrine can reach velocities of up to 350 kmh^−1^ (Tucker 1998). During a dive, peregrine falcons move their wings closer and closer to their body and at top speed they build a wrap dive vacuum pack, i.e., the wings are completely folded against their elongated body. While guarding the nesting site, I often saw that a diving peregrine maintains a remarkable maneuverability. To give an example, during courtship I saw a male falcon dive down in front of a female almost vertically from an estimated height of 1000 m (even with binoculars with eight times magnification, the male peregrine was only a small dot in the sky before beginning its dive) to then make a 180° turn immediately before impact with the ground, bringing the male back to a height of about 100 m (the height of the rock in which the female was sitting) in a few seconds. In fact, my first publication in a popular journal was about peregrine falcons (Bleckmann 1973). I never forgot this amazing experience and one day very many years later, I mentioned my falcon observations to Christoph Brücker (Fig. 9) with whom I had carried out many research projects. As an engineer of fluid mechanics, he immediately got interested in the subject. Together with Benjamin Ponitz (a PhD student of Christoph), Anke Schmitz and Dominik Fischer, who had access to live falcons, we investigated the aerodynamics of a diving peregrine (Ponitz et al. 2014). Furthermore, we studied the morphology and the material properties of the wing and tail feathers of *F. peregrinus*. For comparison, we also investigated the feathers of European kestrels (*F. tinnunculus*), sparrow hawks (*Accipiter nisus*) and pigeons (*Columba livia*). Among others, we found that the wing and tail feathers of *F. peregrinus* had the most striking cross sections and the highest bending stiffness of the species investigated (Schmitz et al. 2014). We also wanted to know the forces that act on the wings of a diving peregrine. Since these forces can hardly be measured directly, we used finite element analysis to estimate these forces. According to our calculations, there is up to 350 g pull on the wings of a peregrine diving with an assumed velocity of 80 ms^−1^ (288 kmh^−1^). During abrupt load changes, these forces are probably substantially higher. Since the wing bones and the shoulder girdle of a diving peregrine experience large mechanical forces, we investigated these bones. Normalized bone mass of the entire arm skeleton and the shoulder girdle was significantly higher in *F. peregrinus* than in *F. tinunculus*, *A. nisus* and *C. livia*. The bones of the arm and shoulder girdle were strongest in peregrine falcons. Furthermore, the midshaft cross section of the humerus of *F. peregrinus* had the highest second moment of area and the bones of the arm and shoulder girdle were strongest in peregrine falcons (Schmitz et al. 2018). As expected, peregrine falcons are well adapted to handle the aerodynamic stresses of extreme flight manoeuvers.

### Biomimetic studies

In addition to the many other research projects, we also worked on the fabrication of biomimetic sensors. When Helmut Schmitz and I started to study the physiology of the IR receptors of *Melanophila*, we had no biomimetic infrared sensors in mind. After having published a paper about the IR organs of *Melanophila* in Nature (Schmitz et al. 1997), we received a letter from the USA. Someone from the US Airforce Office of Scientific Research (AFOSR) invited us to join a multidisciplinary group of scientists who were trying to develop biomimetic IR sensors. The money they (and later also the American Defense Advanced Research Projects Agency (DARPA)) offered not only gave us the opportunity to continue and extend our IR studies, but also to develop and patent a low-cost prototype of an IR sensor, based on the photomechanic IR sensor of *Melanophila* (Bleckmann et al. 2000, 2004).

Inspired by our biomimetic IR sensor, we also worked on the fabrication of biomimetic lateral lines (Yang et al. 2011; Klein and Bleckmann 2011b, a; Herzog et al. Herzog et al. 2015a, 2015b). These studies were also funded by DARPA and by the European Union (EU). One motive for our attempts to build artificial lateral lines were possible industrial applications. Therefore, we showed our biomimetic sensors twice at the Hannover fair (the most important industrial fair in Germany). In Hannover we got many suggestions for what our sensors were suitable for. In addition, a cooperation with an industrial partner came about.

We soon realized that biomimetic lateral lines could help us to learn more about the fish lateral line. During my stay in Ted Bullock’s laboratory, Heinrich Münz from the University of Bielefeld and I had studied the peripheral physiology of the lateral line of the marine teleost *Xiphister atropurpureus*. This teleost inhabits the shoreline of Northern California and Oregon and is often exposed to turbulent water conditions. *X. atropurpureus* has four trunk lateral line canals on each body side instead of the usual one. Furthermore, each canal has side branches called tubuli. Pores are absent in the main canals but each tubulus has between three and five pores. In total, *Xiphister* has several hundred canal pores on each body side, i.e., its trunk looks like a sieve. This led to the question of what advantage the tubuli with their many pores offer. In physiological studies, done 35 years ago in Ted Bullock’s laboratory, we had not been able to uncover the functional significance of the *Xiphister* trunk lateral line (Bleckmann and Münz 1990). However, with the aid of artificial canals, each of which was equipped with one artificial neuromast, we finally were able to find out the filter properties of the *Xiphister* lateral line. Artificial simple canals and a complex *Xiphister*-like canal were exposed to laminar water flow. Upstream to our lateral line canals was a cylinder that shed vortices. In some experiments, hydrodynamic noise, created with small air bubbles, was added. Without noise, the output signals of our artificial canals (artificial lateral line neuromasts) showed periodic peaks at the vortex shedding frequency. If air bubble noise was added, the peaks were smaller and superimposed by less predictable peaks. However, response masking was greater in the simple lateral line canal than in the *Xiphister*-like canal. Obviously, the trunk lateral line canals of *Xiphister* functioned as spatial noise filters (Klein et al. 2013). Together with our biomimetic lateral line sensors, we were now able to build artificial lateral line systems with many different filter properties.

In the context of our biomimetic research, members of my laboratory also studied friction-enhancing microstructures in chameleons, geckos and anguid lizards (Riedel et al. 2015; Spinner et al. 2014, 2015; Jendrian et al. 2014), non-contaminating camouflage in West African Gaboon vipers (Spinner et al. 2014), venom flow in the venom channel of spitting cobras (Triep et al. 2013), and vortex formation by an artificial snapping shrimp claw (Hess et al. 2013).

During my 40 years as active researcher I, together with my students, postdocs, colleagues and collaborators, have worked with teleosts, sharks, marine and freshwater rays, fishing spiders, seals, water rats, amphibians, venomous snakes, pistol shrimps, cephalopods, insects, chameleons, geckos, lizards and birds of prey. The total number of species studied amounts to more than 35. During my time as an active researcher, I learned a lot from my postdocs, colleagues, collaborators and students, but also from the many species we have worked on. Even after 40 years of active research, I still tend to underestimate the sensory and behavioral capabilities of animals. In my opinion only comparative studies have the power to uncover at least a small fraction of the gigantic untapped reservoir of natural solutions for many problems. I admit that the study of model organisms is very useful as exemplified by studies on the fruit fly *Drosophila melanogaster* (Sokolowski 2001), the nematode *Ceanorhabditis elegans* (Kaletta and Hengartner 2006) or transgenic mice (Hickmann-Davis and Davis 2006). The studies of model organisms have provided tremendous insight into how animals function, even at the genetic and molecular level. However, without comparative research, we would never have discovered the IR receptors of *Melanophila* and other fire loving beetles, that seals can use their vibrissae to track fish wakes, that weakly electric fish can discriminate purely Ohmic from capacitive objects or that the *Xiphister* trunk lateral lines acts as a spatial noise filters. We would not know that sharks have cognitive abilities similar to those of mammals and we would be unaware of most of the many different morphological, physiological and behavioral adaptations nature has invented. During my time as an active researcher, I was mostly attracted by non-model organisms, and it is no wonder that 39 of my papers appeared in my home journal, the Journal of Comparative Physiology A. In one of the many conversations I had with Ted Bullock some decades ago in San Diego, he explained to me: “There are two types of biologists. One who knows everything about nothing and one who knows a little bit about everything.” And he added “you and I belong to the second type and we have more fun.”

## Abbreviation

IR: Infrared
